# Classical homocystinuria: A common inborn error of metabolism? An epidemiological study based on genetic databases

**DOI:** 10.1002/mgg3.1214

**Published:** 2020-03-30

**Authors:** Giovana R. Weber Hoss, Fernanda Sperb‐Ludwig, Ida V. D. Schwartz, Henk J. Blom

**Affiliations:** ^1^ BRAIN Laboratory Hospital de Clínicas de Porto Alegre Porto Alegre Brazil; ^2^ Post Graduate Program in Genetics and Molecular Biology Universidade Federal do Rio Grande do Sul (UFRGS) Porto Alegre Brazil; ^3^ Medical Genetics Service Hospital de Clínicas de Porto Alegre Porto Alegre Brazil; ^4^ Department of Clinical Genetics Center for Lysosomal and Metabolic Diseases ErasmusMC Rotterdam The Netherlands

**Keywords:** classical homocystinuria, frequent pathogenic variants, genomic databases, homocysteine, incidence

## Abstract

**Background:**

Biallelic pathogenic variants in *CBS* gene cause the most common form of homocystinuria, the classical homocystinuria (HCU). The worldwide prevalence of HCU is estimated to be 0.82:100,000 [95% CI, 0.39–1.73:100,000] according to clinical records and 1.09:100,000 [95% CI, 0.34–3.55:100,000] by neonatal screening. In this study, we aimed to estimate the minimal worldwide incidence of HCU.

**Methods:**

The 25 most common pathogenic alleles of HCU were identified through a literature review. The incidence of HCU was estimated based on the frequency of these common pathogenic alleles in a large genomic database (gnomAD).

**Results:**

The minimum worldwide incidence of HCU was estimated to be ~0.38:100,000, and the incidence was higher in Europeans non‐Finnish (~0.72:100,000) and Latin Americans (~0.45:100,000) and lower in Africans (~0.20:100,000) and Asians (~0.02:100,000).

**Conclusion:**

Our data are in accordance with the only published metanalysis on this topic. To our surprise, the observed incidence of HCU in Europeans was much lower than those described in articles exploring small populations from northern Europe but was similar to the incidence described on the basis of neonatal screening programs. In our opinion, this large dataset analyzed and its population coverage gave us greater precision in the estimation of incidence.

## Graphical abstract

The 25 most common pathogenic alleles of HCU were identified through a literature review. The incidence of HCU was estimated based on the frequency of these common pathogenic alleles in a large genomic database. The minimum worldwide incidence of HCU was estimated to be ~0.38:100,000. The observed incidence of HCU in Europeans was much lower than those described in articles exploring small populations from northern Europe but was similar to the incidence described on the basis of neonatal screening programs

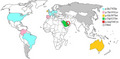

## 1 INTRODUCTION

The most common cause of genetic homocystinuria is cystathionine beta‐synthase (CBS) deficiency or classical homocystinuria (HCU) (OMIM #236200), which is an autosomal recessive disease caused by biallelic pathogenic variations in *CBS* gene. There are also other 13 genes related to monogenic homocystinurias like *MTHFR*, *MMACHC*, and *MMADHC*. HCU is biochemically characterized by the accumulation of homocysteine (Hcy) and methionine (Met) and by decreased cysteine levels. The main clinical complications in untreated HCU patients are found in the eyes, skeleton, central nervous system, and vascular system (Morris et al., 2017).

More than 200 pathogenic variants have been described in the *CBS* gene, and most of these are rare and private variants (Stenson et al., 2017). However, the four most prevalent mutations (p.Ile278Thr, p.Thr191Met, p.Gly307Ser, and p.Trp323Ter) represent half of all HCU alleles reported worldwide (Kraus, 2019). Rare metabolic monogenic diseases like HCU are usually characterized by allelic heterogeneity and show a broad spectrum of clinical expressivity (e.g., for some diseases, even the penetrance is not 100%). Besides that, in the absence of newborn screening programs, their diagnosis is usually delayed. Those factors together contribute for making the estimating of the incidence/prevalence of those diseases a real challenge. In the case of HCU, this is even more complicated, since there is not a good biomarker for newborn screening of the milder forms (the “responsive to pyridoxine” patients).

The worldwide prevalence of HCU based on the number of known patients is estimated to be between 0.29 and 1:100,000 individuals (Moorthie, Cameron, Sagoo, Bonham, & Burton, 2014; S.H., H.L., & J.P., 2001). Moorthie et al., 2014 performed a systematic review and meta‐analysis to estimate the prevalence of HCU and found a worldwide prevalence based on diagnosis of symptomatic individuals of 0.82:100,000 [95% CI, 0.39–1.73:100,000], while that based on neonatal screening by MS/MS was 1.01:100,000 [95% CI, 0.34–3.55:100,000] newborns (NBs). We like to point out that the study of Moorthie et al., 2014 included the Qatari population, with an extremely high prevalence of HCU of 55:100,000 which may introduce an overestimation of the worldwide prevalence.

Another strategy to estimate the incidence of HCU is via determining the frequency of carriers for pathogenic alleles in the *CBS* gene and next use it to calculate the expected number of patients with HCU via the Hardy–Weinberg (HW) equation. The first researchers to use this approach were Gaustadnes, Ingerslev, & Rütiger, 1999, who screened 500 consecutive Danish NBs for the c.833T**>**C mutation and estimated the incidence of HCU to be at least 4.8:100,000. Linnebank et al., 2001 also conducted screening for the c.833T**>**C mutation in 200 healthy unrelated German controls and calculated the frequency of homozygosity for this mutation to be 5.6:100,000 individuals. In Norway, Refsum, Fredriksen, Meyer, Ueland, & Kase, 2004 determined the presence of six specific mutations of the *CBS* gene in 1,133 NB blood samples randomly selected from ~12,000 samples, and they calculated an HCU prevalence of ~15.6:100,000. Janosík et al., 2009 estimated the frequency of HCU in the Czech Republic via determining the presence of the c.1105C**>**T mutation in 600 NB blood spots, and they calculated the birth prevalence for HCU to be at least 2.5:100,000.

Thus, there is an about a 6‐fold unexplained discrepancy between the number of known patients with HCU and that calculated on the basis of the number of carriers detected via genetic analyses of relatively small populations in Northwest Europe. There is no clear explanation for this discrepancy, but it could be due to the low penetrance or expressivity of some genotypes or to underdiagnoses. This discrepancy triggered us to obtain a more reliable estimate of the minimal worldwide incidence of HCU, using the data available in relevant large genomic databases.

## 2 METHODS

We determined the 25 most common pathogenic variants in HCU patients via a literature review using the key terms “*CBS* mutation” and “Classical homocystinuria” in PubMed (www.ncbi.nlm.nih.gov/pubmed) and by examining references cited in related papers. Publications that contained molecular data in HCU patients were selected and used in the analysis, the search resulted in the inclusion of forty papers, containing 1,026 independent alleles from 25 countries. Since several of these studies described only a few patients, which could lead to overestimation of the frequency of a specific allele, only data from those countries in which at least ten alleles (five non‐related patients) were used in the analyses totaling 1,014 alleles (Table 1).

**Table 1:** Frequency of the 25 most prevalent pathogenic *CBS* variants found among HCU published patients worldwide

Country	Allelic frequency												
	Total nº of alleles	p.Arg336Cys	p.Ile278Thr	p.Gly307Ser	p.Thr191Met	p.Trp323Ter	c.1224‐2 A**>**C	p.Asp444Asn	p.Ala114Val	p.Arg125Gln	p.Thr353Met	p.Arg266Lys	p.Lys523Serfs
Anglo‐Celtic (Australia)	50	0.02	0.20	0.22							0.02		
Argentina	18				0.11				0.05				
Brazil	82		0.16		0.15	0.06					0.01		
China	20	0.05	0.05							0.15			
Colombia	34				0.73								
Czech Republic and Slovakia	60		0.20				0.18		0.02				
Denmark	10		0.20					0.20					
England	34	0.03	0.29	0.23						0.03			
France	12		0.17	0.08				0.08					
Germany	12		0.33	0.17									
Ireland	78		0.01	0.66						0.02			
Israel	16		0.18	0.06									
Italy	62		0.29						0.14	0.01			
Japan	24		0.08						0.04				
Korea	10	0.10									0.10 (1/10)		
Norway	32		0.12	0.19								0.34	
Poland	14		0.36										
Portugal	26	0.08	0.04		0.23					0.11			0.23
Qatar	142	0.97		0.01									
Russia	22		0.04				0.27				0.04		
Saudi arabia	26	0.15				0.77							
Spain	80	0.01	0.02		0.44			0.10		0.01	0.05		0.05
The Netherlands	42		0.55					0.05					
Usa	98		0.19	0.07				0.01	0.01	0.02			
Venezuela	10				0.20			0.20					
Total	1,014												

Country	Allelic frequency (cont.)													
	Total nº of alleles	p.Glu144Lys	p.Cys165Tyr	p.Thr257Met	p.Arg121His	p.Val320Ala	p.Gly85Arg	p.Gly151Arg	p.Leu101Pro	p.Thr262Met	p.Gln7Profs	p.Ala226Thr	p.Lys441Ter	p.Gly148Arg
Anglo‐Celtic (Australia)	50	0.10	0.02						0.02					
Argentina	18						0.05					0.22		0.11
Brazil	82			0.01			0.02	0.06						
China	20			0.05										
Colombia	34				0.09									
Czech Republic and Slovakia	60	0.03	0.03								0.05			
Denmark	10						0.10 (1/8)							
England	34	0.03												
France	12													
Germany	12	0.08												
Ireland	78								0.05	0.01				
Israel	16													
Italy	62			0.01										
Japan	24				0.16			0.04					0.16	
Korea	10			0.10										
Norway	32					0.12				0.06				
Poland	14													0.14
Portugal	26													
Qatar	142													
Russia	22													
Saudi Arabia	26													
Spain	80			0.025										
The Netherlands	42		0.09											
USA	98					0.02			0.01	0.02				
Venezuela	10						0.20							
Total	1,014													

Note

Country and the total number of independent alleles analyzed according to studies included in this research. Results for each pathogenic variant are presented as: frequency of allele. The most common pathogenic variant in each country is underlined.

Based on the 25 most frequent variants of the literature review, we conducted searches to determine the prevalence of these variants in the general population in two relevant genomic databases: gnomAD v2.1.1 (Lek et al., 2016, last accessed October 2019) and ABraOM (Naslavsky et al., 2017, last accessed July 2019). The first database includes worldwide data from 141,456 unrelated individuals sequenced as part of various disease‐specific and population genetic studies and it is possible to access different subgroups in which individuals can overlap (e.g., one individual could be included in more than one subgroup): controls, non‐cancer, non‐neuro, and non‐TOPMed. The individuals are clustered according to their genetic determination of ancestry. For example, individuals residing in the USA or Brazil may be clustered as European, African or Asian according to their genetic background. The second database, ABraOM, uses data from 609 healthy elderly individuals who were selected using a standardized sampling process from the city of São Paulo, Brazil; nearly 10% of the Brazilian population is located in this city, making it reasonably representative of the country.

The estimated incidence of HCU was calculated based on the assumption that HW equilibrium exists; thus, the frequencies are ‘‘p’’ for the wild‐type allele and ‘‘q’’ for the pathogenic allele. The different allele frequencies for each pathogenic variant were summed.

## 3 RESULTS

HCU patients from all selected studies were grouped according to their country of origin, and allelic frequencies were calculated for each variant in each country. The 25 most frequent variants of the *CBS* gene are described in Table 1.

### 3.1 Most common variants

**Figure 1:**
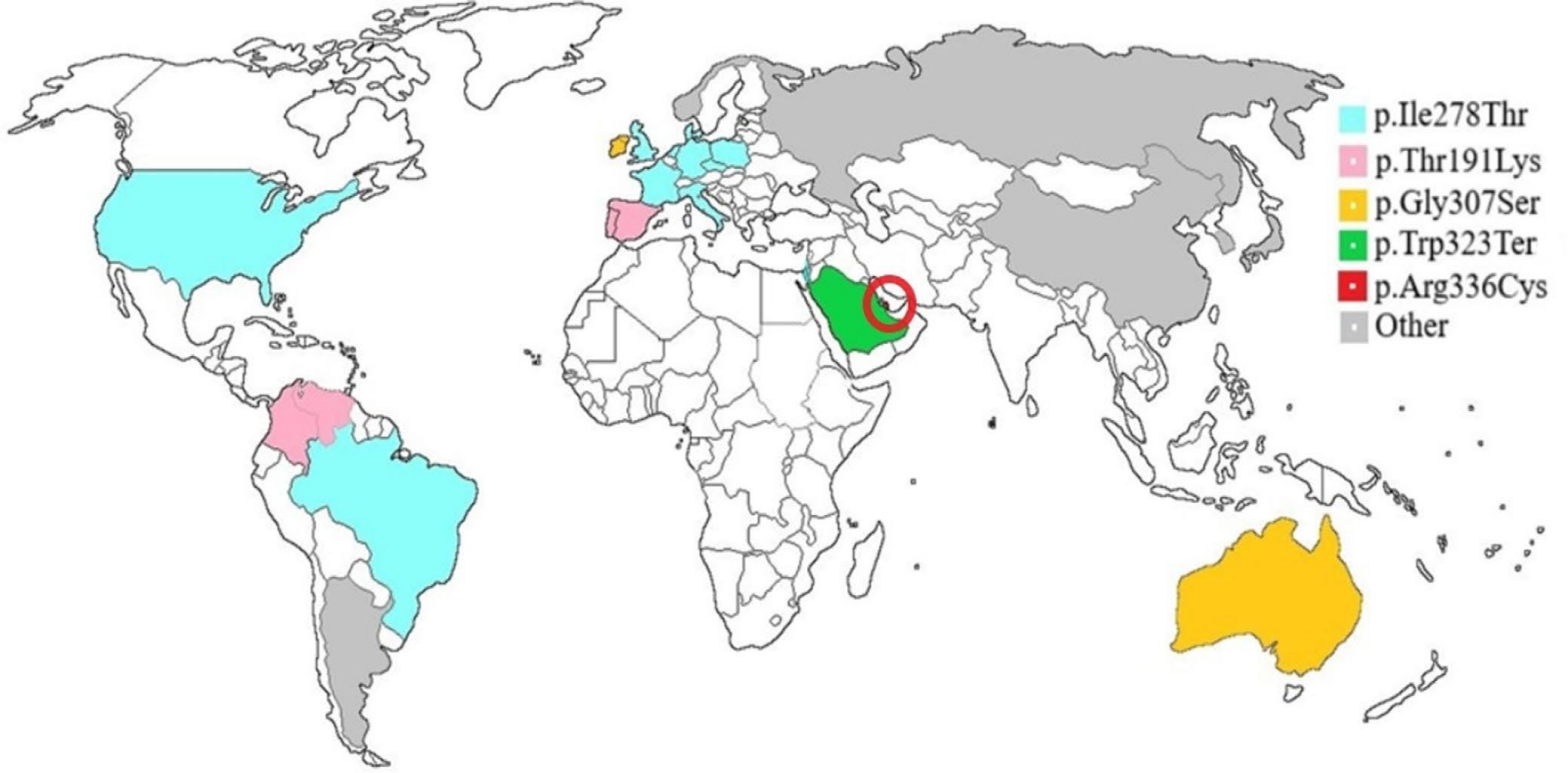
Most common pathogenic alleles of the *CBS* gene per country: p.Ile278Thr, p.Thr191Met, p.Gly307Ser, Trp323Ter, and p.Arg336Cys. The most prevalent variant in the world (p.Ile278Thr) is the most common in The Netherlands (allelic frequency: 55%), Poland (36%), Germany (33%), England (29%), Italy (29%), Denmark (20%), Czech Republic and Slovakia (20%), USA (19%), Israel (18%), France (17%), and Brazil (16%). The variant p.Thr191Met is the most common in Colombia (73%), Spain (44%), Portugal (23%), and Venezuela (20%). In Ireland (66%) and Australia (22%) the most common variant is p.Gly307Ser. The variant Trp323Term is the most common in Saudi Arabia (77%), and in Qatar (highlighted by the red circle) the most common variant is p.Arg336Cys (97%). Other prevalent mutations are c.700_702delGAC in Korea (20%), c.1224‐2A>C in Russia (27%), p.Arg121His and p.Lys441Ter in Japan (16% each one), p.Arg125Gln in China (15%), in Argentina p.Ala226Thr (22%) and in Norway p.Arg266Lys (34%)

The five most common pathogenic *CBS* variants identified in our literature review (46% of alleles) were p.Arg336Cys, p.Ile278Thr, p.Gly307Ser, p.Thr191Met, and p.Trp323Ter. The countries where these pathogenic variants are most common are highlighted in Figure 1 and Table 1.

### 3.2 p.Arg336Cys

In this study, p.Arg336Cys presented an overall allele frequency among HCU patients of 14% (149 alleles), and it was by far the most common variant in Qatar (97% of alleles). p.Arg336Cys was found in 15% of HCU Saudi Arabian patients but in no more than 10% of cases in European and Asian patients.

In the gnomAD, this variant was found only in non‐Finnish Europeans, and it was present in 0.004% of alleles in this population. Patients homozygous for p.Arg336Cys are usually unresponsive to treatment with pyridoxine, and untreated patients present a severe clinical phenotype with involvement of the eyes, bones and vascular and central nervous systems.

### 3.3 p.Ile278Thr

**Figure 2:**
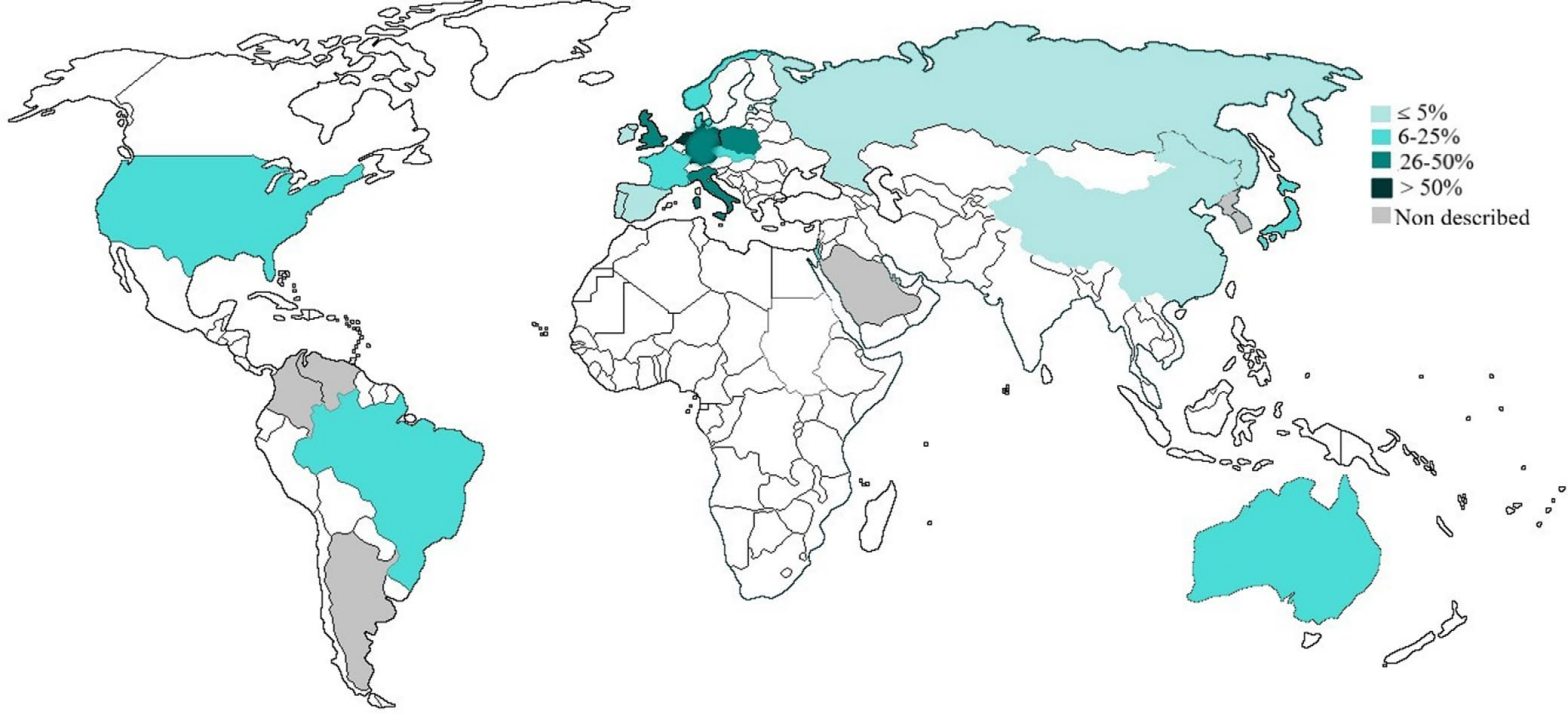
Distribution of the p.Ile278Thr variant among Classical Homocystinuria patients. Presence of the pathogenic variant in countries with at least 10 pathogenic alleles of HCU described in the literature. Countries without at least 10 published HCU alleles, or with no HCU patients genotyped, are presented in white. Gray indicates countries in which the variant p.Ile278Thr was not described among HCU patients

Our data showed an allele frequency among HCU patients of 13% (133 alleles) for p.Ile278Thr, which is the most widely dispersed variant in the world. The p.Ile278Thr was the most common pathogenic variant reported in the USA, Brazil, France, Italy, Germany, the Netherlands, the Czech Republic, Slovakia, Poland, Denmark, England, and Israel. Figure 2 illustrates the presence and frequency of this pathogenic variant around the world in HCU patients.

According to gnomAD, this variant was found in non‐Finnish Europeans (0.143% of alleles were pathogenic), Finnish Europeans (0.057%), and Africans (0.023%), but it was not present in Asian or Latin American individuals; however, it was present in almost 1% of alleles in the Brazilian sample studied in ABraOM. Patients homozygous for p.Ile278Thr are usually responsive to treatment with pyridoxine and present a mild to moderate phenotype.

### 3.4 p.Gly307Ser

A reported allele frequency of 10% (108 alleles) was found for p.Gly307Ser in HCU patients from the USA, Europe, Israel, Australia, and Qatar. p.Gly307Ser was the most common reported pathogenic variant in Ireland (66%) and Australia (22%).

According to gnomAD this pathogenic allele was present in Europeans (0.03% of all alleles) and Africans (0.008%) (Table 2). Patients homozygous for p.Gly307Ser are usually non‐responsive to treatment with pyridoxine and present a severe clinical phenotype.

**Table 2:** Incidence of carriers individuals for each pathogenic variant in the *CBS* gene in different populations according to the genomic database gnomAD total, non‐neuro, and control subgroups

Variant (frequency in total gnomAD population (T); gnomAD non‐neuro (NN) and gnomAD controls (C)		Europeans non‐Finnish Alleles (*n*): T:129,158; NN:103,136; C:48,282	Europeans Finnish Alleles (*n*): T: 25,188; NN: 17,846; C: 14,504	Africans Alleles (*n*): T: 24,968; NN: 19,600; C: 9,736	Latin Americans Alleles (*n*): T: 35,436; NN: 31,074; C:17,354	Asian Alleles (*n*): T: 50,568; NN: 45,582; C: 25,652	Ashkenazi Jewish Alleles (*n*): T: 10,370; NN: 6,458; C: 2,358	Others Alleles (*n*): T: 7,224; NN: 5,712; C: 2,406	TOTAL^a^ (allele frequency) Alleles (*n*): 282,912	NON‐NEURO GROUP (allele frequency) Alleles (*n*) 229,408	CONTROL GROUP ONLY (allele frequency) Alleles (*n*) 120,292
p.Arg336Cys (c.1006 C>T)	T: 0.00004417; NN: 0.00003365; C: 0.00002341		—	—	—	—	—	—	0.00001994	0.00001445	0.00000915
p.Ile278Thr (c.833 T>C)	T: 0.00143200; NN: 0.00154900; C: 0.00091170		T: 0.00057600 NN: — C: —	T: 0.00023230; NN: — C: —	—	—	—	—	0.00083270	0.00099240	0.00046250.
p.Gly307Ser (c.919 G>A)	T: 0.00031740; NN: 0.00037810; C: 0.00031070		T: 0.00003989; NN: 0.00005603; C: 0.00006895	T: 0.00008010; NN: 0.00010200; C: 0.00020540	—	—	—	T: 0.00041530; NN: 0.00035740; C: 0.00042810	0.00016620	0.00019190	0.00015800
p.Thr191Met (c.572 C>T)	—		—	—	T: 0.00037700; NN: 0.00036150; C: 0.00023440	—	—	0.00032890; 0.00020680	0.00006023	0.00005790	0.00003663
p.Trp323Ter (c.969 G>A)	—		—	—	—	T: 0.00003270; NN: 0.00003271; C: 0.00006380	—	—	0.00000399	0.00000482	0.00000915
c.1224‐2 A>C	T: 0.00004088; NN: 0.00002584; C: 0.00002739		—	—	—	—	T: 0.00138800; NN: 0.00162200; C: 0.00051230	—	0.00007850	0.00006121	0.00002173
p.Asp444Asn (c.1330 G>A)	T: 0.00026390; NN 0.00026010; C 0.00031750		T: 0.00005424 NN: — C: —	T: 0.00021710; NN: 0.00022950; C: 0.00033460	T: 0.00126600; NN: 0.00066670; C: 0.00067920	—	—	T: 0.00052580; NN: 0.00022420; C: 0.00053940	0.00032120	0.00022680	0.00025970
p.Ala114Val (c.341 C>T)	T: 0.00035680; NN: 0.00039820; C: 0.00033150		—	T: 0.00008024; NN: 0.00010220; C: 0.00010280	T: 0.00028230; NN: 0.00032190; C: 0.00051870	T: 0.00013296; NN: 0.00016637; C: 0.00010040	—	—	0.00021600	0.00024460	0.00022490
p.Arg125Gln (c.374 G>A)	T: 0.00002326; NN: 0.00001942; C: 0.00018180		—	—	—	—	—	—	0.00001062	0.00000874	0.00009213
p.Thr353Met (c.1058 C>T)	—		—	T: 0.00029160; NN: 0.00039310; C: 0.00014530	T: 0.00002840; NN: 0.00003299; C: —	—	—	—	0.00002973	0.00003571	0.00000988
p.Arg266Lys (c.797 G>A)	T: 0.00001772; NN: 0.00001125; C: 0.00002345		—	—	—	—	—	—	0.00000801	0.00000484	0.00000918
p.Lys523Serfs (c.1566delG)	—		—	—	—	—	—	—	—	—	—
p.Glu144Lys (c.430 G>A)	T: 0.00003882; NN: 0.00004863; C: 0.00002340		—	T: 0.00004020; NN: 0.00005118; C: 0.00014010	—	T: 0.00009799; NN: 0.00009802; C: 0.00006374	—	—	0.00003198	0.00003947	0.00002767
p.Cys165Tyr (c.494 G>A)	—		—	—	—	—	—	—	—	—	—
p.Thr257Met (c.770 C>T)	T: 0.00001564; NN: 0.00001956; C: 0.00002084		—	T: 0.00004065; NN: 0.00005195; C: 0.00010540	T: 0.00011340; NN: 0.00009706; C: 0.00011580	T: 0.00013230; NN: 0.00013230; C: 0.00006450	—	T: 0.00013960; NN: 0.00018030; C: —	0.00004286	0.00004840	0.00004191
p.Arg121His (c.362 G>A)	T: 0.00002325; NN: 0.00001942; C: —		—	T: 0.00024090; NN: 0.00030650; C: 0.00041110	—	—	—	—	0.00003187	0.00003494	0.00003332
p.Val320Ala (c.959T>C)	T: 0.00003539; NN: 0.00004496; C: 0.00007028		—	—	—	—	—	—	0.00001598	0.00001930	0.00002747
p.Gly85Arg (c.253 G>A)	T: 0.00000879; NN: 0.00001117; C: 0.00002338		—	—	—	—	—	—	0.00000398	0.00000481	0.00000914
p.Gly151Arg (c.451 G>A)	T: 0.00002335; NN: 0.00002926; C: —		—	T: 0.00008055; NN: — C: —	T: 0.00002825; NN: 0.00003221; C: —	—	—	—	0.00002142	0.00001764	—
p.Leu101Pro (c.302 T>C)	—		—	—	—	—	—	—	—	—	—
p.Thr262Met (c.785C>T)	T: 0.00001772; NN: 0.00002251; C: —		T: 0.00014060; NN: 0.00018040; C: 0.00022560	T: 0.00006242; NN: 0.00006257; C: —	—	—	—	T: 0.00016400; NN: 0.00020700 C: —	0.00002804	0.00003387	0.00002757
p.Gln7Profs (c.19dupC)	—		—	—	—	T: 0.00005473; NN: 0.00013130; C: 0.00006401	—	—	0.00001645	0.00000498	0.00000922
p.Ala226Thr (c.676G>A)	—		—	—	—	—	—	—	—	—	—
p.Lys441Ter (c.1321A>T)	—		—	—	—	—	—	—	—	—	—
p.Gly148Arg (c.442G>A)	T: 0.000008825; NN: 0.00001122; C: 0.00002341		—	T: 0.00006176; NN: 0.00006190; C: —	T: 0.00002893; NN: 0.00003278; C: 0.00005849	—	—	—	0.00001202	0.00001455	0.00001853
TOTAL	T: 0.00267672; NN: 0.00288229; C: 0.00228876		T: 0.00085693; NN: 0.00023643; C: 0.00029455	T: 0.00142782; NN: 0.00136090; C: 0.00144470	T: 0.00212428; NN: 0.00154514; C: 0.00160659	T: 0.00045068; NN: 0.00056070; C: 0.00035645	T: 0.00138800; NN: 0.00162200; C: 0.00051230	T: 0.0015736; NN: 0.00117570; C: 0.00096750	0.00195171	0.00206131	0.00148778
Incidence of HCU per 100,000 individuals	T: ~ 0.72; NN: ~0.83; C: ~0.52		T: ~ 0.07; NN: ~0.005; C: ~0.008	T: ~ 0.20; NN: ~0.18; C: ~0.21	T: ~ 0.45; NN: ~0.24; C: ~0.26	T: ~ 0.02; NN: ~0,03; C: ~0.01	T: ~0.19; NN: ~0.26; C: ~0.02	T: ~0.25; NN: ~0.14; C: ~0.09	~ 0.38	~0.42	~0.22

Note

—, no alleles found.

^a^ The total population in gnomAD (T) includes the following subgroups, which may overlap: controls (C), non‐cancer, non‐neuro (NN) and non‐TOPMed.

### 3.5 p.Thr191Met

p.Thr191Met presented an allele frequency among HCU patients of 8% (82 alleles) and was the most common pathogenic variant reported in countries of the Iberian Peninsula and in their former colonies in Latin America. The highest frequencies of this variant among HCU patients were found in Spain (44% of the alleles), Portugal (23%), Colombia (73%), and Venezuela (20%).

Data from gnomAD indicated the presence of the variant in Latin Americans (0.038%), but it was not identified in ABraOM. Patients who are p.Thr191Met homozygous are usually non‐responsive to pyridoxine and present a moderate to severe clinical phenotype.

### 3.6 p.Trp323Ter

The overall allele frequency among HCU patients of p.Trp323Ter was 2% (25 alleles). This variant was reported in patients from Saudi Arabia (77% of alleles) and northeast Brazil (6% of alleles). Interestingly, according to gnomAD data this variant is very rare and found only in one allele among Asians. This variant was not observed in ABraOM, which analyzed persons from São Paulo, Brazil. Patients homozygous for p.Trp323Ter are usually non‐responsive to treatment with pyridoxine and present a moderate to severe clinical phenotype.

### 3.7 HCU worldwide incidence

In the genetic database gnomAD, we found 304 individuals who were carriers for any of 20 of the 25 most frequent pathogenic alleles of the *CBS* gene, yielding an estimated HCU incidence (i.e., homozygosity or compound heterozygosity) of ~0.38:100,000 (95% CI, 0.29–0.39:100,000) individuals. When we analyzed only the control group of gnomAD, the estimated incidence was ~0.22:100,000; if we analyze only the non‐neuro subgroup the incidence is ~0.42:100,000 in (Table 2). No homozygous individuals, for the 25 most frequent pathogenic variants, were found in this database.

Analyzing the data according to different ancestry, we calculated an HCU incidence of ~0.72:100,000 individuals among Europeans (non‐Finnish), ~0.45:100,000 individuals among Latin Americans, ~0.20:100,000 individuals among Africans and ~0.02:100,000 individuals in Asians (Table 2).

### 3.8 HCU incidence in southeastern Brazil

In the ABraOM database, we found only two of the 25 variants analyzed (p.Ile278Thr and p.Ala114Val). A total of 12 individuals carrying either of the pathogenic variants were included in this database (11 carriers of p.Ile278Thr and one of p.Ala114Val), yielding an estimated incidence of HCU of ~9.7:100,000 individuals. No homozygous individuals were found.

## 4 DISCUSSION

Knowledge of the genetic background of HCU in different populations is generally poor and even contradictory. This omission hampers proper patient genetic counseling and appropriate genetic testing. Knowledge of the prevalent pathogenic variants and their frequencies will support decision making within national screening programs. Furthermore, there is an approximately 6‐fold discrepancy between the number of known patients with HCU and the estimate calculated on the basis of the number of heterozygotes detected via genetic analyses of relatively small populations.

In this study, we used the results of published articles to characterize the worldwide mutational profiles of HCU patients. Next, we used the 25 most common published pathogenic variants (Table 1) to determine the corresponding allele frequencies in genomic databases and to calculate the incidence of HCU in different ancestralities (Table 2). Interestingly, the frequencies of these 25 most commonly pathogenic variants reported in various countries are in line with the data from genomic databases; for instance, p.Ile278Thr was very common in different ancestries, p.Thr257Met and p.Ala114Val were described in patients with HCU in different continents and found in several ancestralities and p.Thr191Met was found in Latin Americans. Europeans seems to be the group with the greatest allelic diversity, which leads us to hypothesize that dispersion of these pathogenic alleles occurred during the colonization period of America and Africa. In addition to the much lower incidence of HCU, we observed a distinct pattern of mutations in Asia and Russia, where 75% of alleles differ from the 10 most common pathogenic variants worldwide.

Based on biochemical neonatal screening data obtained by the measurement of Met in dried blood spots (DBSs), Naughten, Yap, & Mayne, 1998 reported HCU incidences of 0.77:100,000 NBs in Germany, 0.8:100,000 NBs in England and 0.34:100,000 NBs in the USA and higher incidences of 1.5:100,000 NBs in Ireland and 1.8:100,000 NBs in Italy. According to Mathias & Bickel, 1986, the incidence in Germany was 0.32:100,000 NBs based on biochemical neonatal screening data of almost 1 million individuals. Biochemical neonatal screening of 820,797 individuals in New South Wales, Australia, around the 1960s revealed 14 cases of HCU, resulting in an incidence of 1.72:100,000 NBs (Wilcken & Turner, 1978).

In Asian countries, a much lower HCU incidence is observed. In Japan, an extremely low incidence of 0.11:100,000 NBs was observed despite an effective biochemical screening program (Naughten et al., 1998). National biochemical neonatal screening performed in the Philippines between 1996 and 2001 identified no HCU patients among 176,548 samples (Padilla, 2003). In Taiwan, 5 million individuals were subjected to biochemical neonatal screening for HCU, and only 3 were diagnosed with the disease. In sharp contrast, an extremely high frequency of HCU of 416:100,000 individuals was found on an island inhabited by an Austronesian Taiwanese Tao tribe (Lu et al., 2012). Kaur, Das, & Verma, 1994 investigated 2,560 high‐risk patients with strong suspicion of an inborn error of metabolism in northern India, and the most commonly found disorder was HCU (0.6%).

Qatar is the country with the highest incidence of HCU in the world due to a founder effect of p.Arg336Cys. This pathogenic variant rate is very frequent in three tribes of the Qatari population, and consanguineous marriages even enhance the high incidence of HCU. Initially the incidence of HCU was estimated to be ~33:100,000 individuals (El‐Said et al., 2006). However, after the implementation of neonatal screening through the detection of tHcy and Met combined with genetic screening, the estimated incidence of HCU in the Qatari population increased to 55:100,000 NBs (Gan‐Schreier et al., 2010).

Newborn screening is being carried out in countries with high incidences of HCU, such as Ireland and Qatar (Yap & Naughten, 1998; Zschocke et al., 2009). For this purpose, tHcy is measured in DBSs with a dedicated method in Qatar. However, all other newborn screening programs measure Met in DBSs, which results in a high proportion of false negatives, particularly for pyridoxine‐responsive forms of HCU, because these patients seem not to develop hypermethioninemia in the first days of life, so due to the limitation of newborn screening method this patients are likely not diagnosed (Bowron, Barton, Scott, & Stansbie, 2005; McHugh et al., 2011; Peterschmitt, Simmons, & Levy, 1999). Countries in Latin America have no neonatal screening program for HCU; however, based on our estimation of HCU incidences in these populations of ~0.45:100,000 we like to advocate to introduce newborn screening for HCU in Latin American countries. Countries such as Japan and the USA have neonatal screening programs for HCU, even though the incidences in these countries are lower than (Japan) or similar to (USA) those estimated in Latin America.

Among *CBS* mutations, p.Ile278Thr is geographically the most widespread. Studying the emergence and dispersal of this mutation, Vyletal et al., 2007 reported that haplotype c. [833C; 844_845ins68] is very common in sub‐Saharan Africa (up to 40% of control chromosomes), less frequent throughout Europe and America (5%–10% of control chromosomes), and rare in Asia (0.16%–2.5% of control chromosomes). It was concluded that the p.Ile278Thr variant occurred repeatedly and independently in the recent history of the European population. Interestingly and confusing is the haplotype c.[833C; 844_845ins68] on the *CBS* gene, which contains the c.833T**>**C. But this haplotype is considered non‐pathogenic since c.844_845ins68 creates an alternative splice site that rescues the wildtype *CBS* sequence from the mutated allele, resulting in normal CBS enzyme activity and normal Hcy concentrations (Kluijtmans et al., 1997). We have to take into account that in the Brazilian database the presence of variant c.844_845ins68 has not been described, but since the frequency of p.Ile278Thr is very high (0.9%), we cannot rule out the possibility that some of these individuals may carry the non‐pathogenic haplotype. In the gnomAD, we assumed that the individuals heterozygous for p.Ile278Thr (0.08% of the total sample) had the isolated pathogenic variant, since the frequency of individuals with the c.844_845ins68 variant was approximately 12%.

The prevalence of HCU varies dramatically between regions from 416:100,000 on Orchid Island and 55:100,000 in Qatar to less than one in one million in the Taiwanese Han population (Gan‐Schreier et al., 2010; Lu et al., 2012). In this study, we used the genetic database gnomAD to determine the frequency of *CBS* heterozygotes and next calculated the worldwide incidence of HCU, which was found to be approximately 0.38:100,000 individuals. Stratifying populations by ancestry, the highest incidence of HCU was found in Europeans and Latin Americans. A much lower incidence was found in Asians. The incidences in these various regions were more or less in line with those found through neonatal biochemical screening. For instance, in Europeans, an incidence of 0.72:100,000 individuals was calculated on the basis of the number of heterozygotes in gnomAD versus 0.77:100,000 according to neonatal screening, and in Asian, the corresponding values were 0.02:100,000 versus 0.07:100,000 individuals (Naughten et al., 1998).

Another remarkable finding is that the incidence calculated in this study for Europeans of approximately ~0.72:100,000 individuals is much lower (approximately 6 to 7 times) than those described in four different studies. At least 4.8:100,000 live births in Denmark, 5.6:100,000 in Germany, 2.5:100,000 in Czech Republic ~15.6:100,000 in Norway. We have no explanation for this discrepancy except that the numbers of studied individuals were relatively small (200 to maximal 1,133 individuals) and that publication bias may have played a role. The Europeans sample size of gnomAD is approximately 115 times larger than the sample sizes of these studies and should therefore provide a much more precise incidence rate.

We estimated the number of HCU patients using HW equilibrium. The HW principle presents limitations because it analyzes allele frequencies and genotype counts in successive generations and predicts that in a random mating population of infinite size, allele and genotype frequencies should remain constant from one generation to the next. Factors that may disrupt the HW equilibrium included mutation rate, natural selection, migration, population structure (nonrandom marriage and/or consanguinity) and nonrandom selection of the samples studied (Piel et al., 2016; Waples, 2015). Overall, we assume that these limitations do not substantially affect the numbers we calculated.

The genomic database gnomAD consists of 282,912 alleles and includes data from consortia such as 1,000 genomes, GO‐ESP and TOPMed and provides sequence data from unrelated individuals from various disease‐specific populations included in genetic studies. Therefore, our frequency analysis is based on diverse populations from various countries and ethnicities clustered according to their genetic determination of ancestry. Although the calculated frequency of HCU is relatively low, we consider it to be rather precise estimation of incidence because of the large number of individuals and the different genetic backgrounds included.

Genetic data provided by databases present limitations because of the heterogeneous inclusion criteria of the original studies such as age or selection based on diseases. Although gnomAD is the largest public genetic database to our knowledge, it should be taken into account that individuals in this database are clustered according to their genetically determined ancestry and not according to the country or continent where they reside. Approximately 45% of individuals are classified as exhibiting European ancestry, so gnomAD only partially reflects global genetic diversity.

Another possible limitation of this study was the method used to define the 25 most common pathogenic variants among HCU patients. We have considered studying all variants described in the *CBS* gene in the gnomAD population, but the filtering of truly pathogenic variants is rather poor and inaccurate, and we kept in mind that the low penetrance or expressivity of some genotypes, could lead to a falsely increased incidence result. So, an extensive review of the literature was performed to identify the most common pathogenic variants in HCU patients, but some relatively common variants may still have been missed. Each study presents its own methodology for the inclusion and diagnosis of patients. In many countries, indicated in white in Figure 1, there are no reported HCU patients. No alleles were found in the genomic databases for five of the twenty‐five pathogenic variants analyzed (p.Leu101Pro, p.Cys165Tyr, p.Ala226Thr, p.Lys441Ter, and p.Lys523Serfs). Our study included three pathogenic variants that are known to be responsive to pyridoxine (p.Arg266Lys, p.Ala226Thr, and p.Ile278Thr), which accounted for an important percentage of the alleles present in this population (~20%). Pyridoxine‐responsive patients are known to present with a milder clinical phenotype and presenting later in life or may even have no symptoms at all.

When analyzing the total population of gnomAD we found an estimated incidence of HCU of 0.38:100,000 individuals. In addition, we also analyzed the subgroups of individuals classified as non‐neuro and controls, and calculated for them the incidence of HCU of 0.42 and 0.22:100,000, respectively. Although the incidence estimations are not exactly the same, it remains more or less similar to those found in neonatal screening and much lower than the incidence reported in other studies with heterozygous analysis.

The genetic database ABraOM consists of 1,218 alleles from individuals living in São Paulo, Brazil, and 12 pathogenic alleles were found in this database. This number resulted in a calculated incidence of HCU of ~9.7:100,000 individuals, which is more in line with the incidence found in the small European studies. If ABraOM is representative of the Southeast Region of Brazil, this result may indicate that many patients are not being diagnosed. However, the relatively small sample size may affect the results. Among the *CBS* mutations reported in Brazilian HCU patients, the variant p.Ile278Thr was the most common (18%) (Poloni et al., 2018). In our search in the genomic database ABraOM, p.Ile278Thr was the most common variant with a striking frequency of 0.00903. It may well be possible that also the haplotype c.[833C; 844_845ins68] was ranked as p.Ile278Thr and so may have resulted in an overestimation of the number of HCU patients.

Since HCU is a treatable disease and given the severe clinical complications of HCU, such as thromboembolic events, dislocation of the lens and neurological complications, the early recognition of HCU patients by health professionals is extremely relevant. The results presented in this study based on the number of heterozygotes for *CBS* pathogenic variants in a large genomic database reinforce efforts to recognize CBS patients and to implement effective newborn screening methods, particularly in regions with high incidences.

## Supporting information

Table S1

## Data Availability

The data that support the findings of this study are openly available in GnomAD at https://gnomad.broadinstitute.org/, the ninth reference; and ABraOM at http://abraom.ib.usp.br/, the sixteenth reference.
