# Supplementary material for: Classical homocystinuria: A common inborn error of metabolism? An epidemiological study based on genetic databases
Source: Mol Genet Genomic Med. 2020 Mar 30;8(6):e1214. doi: 10.1002/mgg3.1214 (PMC7284035; doi:10.1002/mgg3.1214)
Supplement: Supplementary file 1 — Table S1 [file MGG3-8-e1214-s001.docx]

| **Supplemental Table: Pathogenic variants reported in the literature in *CBS* gene.** | | | | | | |
| --- | --- | --- | --- | --- | --- | --- |
|  | **Location** | **Nº of Alleles** | **Codon** | **Protein Change** | **References** | **Countries** |
| 1 | 5 prime UTR | 1 | c.-541_-532del | p.(=) | (Urreizti et al., 2006) | Argentina. |
| 2 | Exon 1 | 1 | c.2T>C | p.Met1Thr | (Poloni et al., 2018) | Brazil. |
| 3 | Exon1 | 5 | c.19dupC | p.Gln7Profs | (Gaustadnes et al., 2002; Janosík et al., 2001) | Australia; Czech Republic and Slovakia. |
| 4 | Exon1 | 2 | c.28delG | p.Val10Trpfs | (Janosík et al., 2001; Poloni et al., 2018) | Brazil; Czech Republic and Slovakia. |
| 5 | Exon 1 | 1 | c.52C>T | p.Arg18Cys | (Lee et al., 2005) | Korea |
| 6 | Exon 1 | 1 | c.129G>A | p.Trp43Ter | (Katsushima et al., 2006) | Japan |
| 7 | Exon 1 | 3 | c.146C>T | p.Pro49Leu | (Cozar et al., 2011; Gaustadnes et al., 2002; Poloni et al., 2018) | Australia; Brazil; Spain. |
| 8 | Exon 1 | 1 | c.172C>T | p.Arg58Trp | (de Franchis, Kraus, Kozich, Sebastio, & Kraus, 1999) | Italy |
| 9 | Exon 1 | 1 | c.194A>G | p.His65Arg | (Janosík et al., 2001) | Czech Republic |
| 10 | Intron 1 | 1 | c.209+1delG | p.(=) | (Poloni et al., 2018) | Brazil. |
| 11 | Intron 1 | 3 | c.209+1G>A | p.(=) | (Gaustadnes et al., 2002; Urreizti et al., 2003) | Australia; Brazil; Spain. |
| 12 | Intron 1 | 1 | c.209dupC | p.(Ala71Glyfs*34) | (Gaustadnes et al., 2002) | Australia. |
| 13 | Intron 1 | 1 | c.210-1G>C | p.(=) | (Janosík et al., 2001) | Czech Republic. |
| 14 | Exon 2 | 1 | c.215A>T | p.Lys72Ile | (Li et al., 2018) | China |
| 15 | Exon 2 | 1 | c.216_217delAT | p.(Lys72Asnfs*32) | (Voskoboeva et al., 2018) | Russia. |
| 16 | Exon 2 | 6 | c.253G>A | p.Gly85Arg | (Cozar et al., 2011; De Lucca & Casique, 2004; Maclean et al., 2002; Poloni et al., 2018) | Argentina; Brazil; Denmark; Venezuela. |
| 17 | Exon 2 | 1 | c.262C>T | p.Pro88Ser | (Sebastio et al., 1995) | Italy |
| 18 | Exon 2 | 2 | c.284T>C | p.Ile95Thr | (Poloni et al., 2018) | Brazil |
| 19 | Exon 2 | 6 | c.302T>C | p.Leu101Pro | (Gallagher et al., 1998; Gaustadnes et al., 2002; Kruger, Wang, Jhee, Singh, & Elsas, 2003) | Australia; Ireland; USA. |
| 20 | Exon 2 | 1 | c.304A>C | Lys102Gln | (Gat-Yablonski, Mandel, Fowler, Taleb, & Sela, 2000) | Israel. |
| 21 | Exon 3 | 2 | c.325T>C | p. Cys109Arg | (Gaustadnes et al., 2002; Voskoboeva, Semyachkina, Yablonskaya, & Nikolaeva, 2018) | Australia; Russia. |
| 22 | Exon 3 | 1 | c.329A>T | p.Glu110Val | (Poloni et al., 2018) | Brazil. |
| 23 | Exon 3 | 13 | c.341C>T | p.Ala114Val | (de Franchis et al., 1999; Janosík et al., 2001; Katsushima et al., 2006; Moat et al., 2004; Sebastio et al., 1995; Sperandeo et al., 1995; Urreizti et al., 2006) | Argentina; Czech Republic and Slovakia Italy; Japan; USA. |
| 24 | Exon 3 | 1 | c.346G>A | p.Gly116Arg | (Li et al., 2018) | China |
| 25 | Exon 3 | 2 | c.361C>T | p.Arg121Cys | (Cozar et al., 2011; Katsushima et al., 2006) | Japan; Spain. |
| 26 | Exon 3 | 7 | c.362G>A | p.Arg121His | (Katsushima et al., 2006; Urreizti et al., 2006) | Colombia; Japan. |
| 27 | Exon 3 | 2 | c.373C>T | p.Arg125Trp | (Kluijtmans et al., 1999; Urreizti et al., 2003) | Spain; The Netherlands. |
| 28 | Exon 3 | 13 | c.374G>A | p.Arg125Gln | (Castro et al., 2001; Cozar et al., 2011; Gaustadnes et al., 2002; Li et al., 2018; Marble, Geraghty, de Franchis, Kraus, & Valle, 1994; Moat et al., 2004; Sebastio et al., 1995) | China; England; Ireland; Italy; Portugal; Spain; USA. |
| 29 | Exon 3 | 1 | c.384G>C | p.Glu128Asp | (Coudé, Aupetit, Zabot, Kamoun, & Chadefaux-Vekemans, 1998) | France |
| 30 | Exon 3 | 1 | c.407T>C | p.Leu136Pro | (Gong et al., 2015) | China |
| 31 | Exon 3 | 1 | c.415G>A | p.Gly139Arg | (Shih et al., 1995) | Italy |
| 32 | Exon 3 | 1 | c.429C>G | p.Ile143Met | (Orendáè et al., 2004) | Poland |
| 33 | Exon 3 | 9 | c.430G>A | p.Glu144Lys | (Gaustadnes et al., 2002; Janosík et al., 2001; Moat et al., 2004; Shih et al., 1995) | Australia; Czech Republic and Slovakia; England; Germany. |
| 34 | Exon 3 | 2 | c.434C>T | p.Pro145Leu | (Urreizti et al., 2006) | Spain. |
| 35 | Exon 3 | 5 | c.442G>A | p.Gly148Arg | (Katsushima et al., 2006; Orendáè et al., 2004; Urreizti et al., 2006) | Argentina; Japan; Poland. |
| 36 | Exon 3 | 1 | c.444dupG | p.(Asn149Glufs*39) | (Gaustadnes et al., 2002) | Australia. |
| 37 | Exon 3 | 2 | c.444delG | p.(Asn149Thrfs*12) | (Poloni et al., 2018) | Brazil. |
| 38 | Exon 3 | 6 | c.451G>A | p.Gly151Arg | (Katsushima et al., 2006; Poloni et al., 2018) | Brazil; Japan. |
| 39 | Intron 3 | 2 | c.451+1G>A | p.(=) | (Li et al., 2018) | China. |
| 40 | Exon 4 | 3 | c.456C>G | p.Ile152Met | (Kluijtmans et al., 1999) | The Netherlands. |
| 41 | Exon 4 | 1 | c.457G>A | p.Gly153Arg | (Zaidi et al., 2012) | Saudi Arabia. |
| 42 | Exon 4 | 1 | c.461T>A | p. Leu154Gln | (Lee et al., 2005) | Korea. |
| 43 | Exon 4 | 1 | c.464C>T | p.Ala155Val | (Lee et al., 2005) | Korea. |
| 44 | Exon 4 | 2 | c.463G>A | p.Ala155Thr | (Janosík et al., 2001) | Czech Republic. |
| 45 | Exon 4 | 1 | c.473C>T | p.Ala158Val | (Gong et al., 2015) | China. |
| 46 | Exon 4 | 8 | c.494G>A | p.Cys165Tyr | (Gaustadnes et al., 2002; Janosík et al., 2001; Kluijtmans et al., 1999) | Australia; Czech Republic and Slovakia; South Africa; The Netherlands. |
| 47 | Exon 4 | 1 | c.493_514del | p.(Cys165Argfs*2) | (Gaustadnes et al., 1998) | Denmark. |
| 48 | Exon 4 | 1 | c.503T>C | p.Val168Ala | (Porto et al., 2005) | Brazil. |
| 49 | Exon 4 | 1 | c.517A>G | p.Met173Val | (Urreizti et al., 2006) | Spain. |
| 50 | Exon 4 | 2 | c.518_520del | p.(Met173del) | (Cozar et al., 2011) | India. |
| 51 | Exon 4 | 3 | c.526G>A | p.Glu176Lys | (Janosík et al., 2001; Kozich et al., 1997) | Czech Republic and Slovakia. |
| 52 | Intron 4 | 1 | c.532-31del29 | p.(=) | (Gat-Yablonski et al., 2000) | Israel. |
| 53 | Intron 4- Intron 6 | 1 | c.532-37_736+438del794 | p.? | (Cozar et al., 2011) | Portugal. |
| 54 | Exon 5 | 3 | c.536_553del18 | p.Asp179_Leu184del | (Gaustadnes et al., 2002) | Australia. |
| 55 | Exon 5 | 1 | c.539T>C | Val180Ala | (Kluijtmans et al., 1999) | The Netherlands; |
| 56 | Exon 5 | 82 | c.572C>T | p.Thr191Met | (Bermúdez et al., 2006; Cozar et al., 2011; De Lucca & Casique, 2004; Poloni et al., 2018; Porto et al., 2005; Urreizti et al., 2006; Urreizti et al., 2003) | Argentina; Brazil; Colombia; Portugal; Spain; Venezuela. |
| 57 | Exon 5 | 1 | c.599C>T | p.Pro200Leu | (Cozar et al., 2011) | Spain. |
| 58 | Exon 5 | 2 | c.604_606del | p.(Ser202del) | (Li et al., 2018) | China. |
| 59 | Intron 5 | 1 | c.667-14_667-7delCTCTTTCT | p.= | (Cozar et al., 2011) | Argentina. |
| 60 | Exon 6 | 1 | c.650C>T | p.Ser217Phe | (Katsushima et al., 2006) | Japan. |
| 61 | Exon 6 | 5 | c.676G>A | p.Ala226Thr | (Cozar et al., 2011; Kruger et al., 2003; Urreizti et al., 2006) | Argentina; USA. |
| 62 | Exon 6 | 2 | c.683A>G | p. Asn228Ser | (Kruger et al., 2003) | USA. |
| 63 | Exon 6 | 3 | c.684C>A | p.Asn228Lys | (Gaustadnes et al., 2002; Orendáè et al., 2004) | Australia; Poland. |
| 64 | Exon 6 | 1 | c.684C>G | p. Asn228Lys | (Gallagher et al., 1998) | Ireland. |
| 65 | Exon 6 | 1 | c.689T>A | p. Leu230Gln | (Li et al., 2018) | China. |
| 66 | Exon 6 | 2 | c.689delT | p.(Leu230Argfs*39) | (Cozar et al., 2011) | Argentina. |
| 67 | Exon 6 | 2 | c.691G>C | p. Ala231Pro | (Poloni et al., 2018; Kruger et al., 2003) | Brazil; USA |
| 68 | Exon 6 | 2 | c.694C>G | p. His232Asp | (Katsushima et al., 2006) | Japan. |
| 69 | Exon 6 | 4 | c.700G>A | p.Asp234Asn | (De Lucca & Casique, 2004; El-Said et al., 2006) | Qatar; Venezuela. |
| 70 | Exon 6 | 2 | c.700_702del | p.(Asp234del) | (Lee et al., 2005) | Korea. |
| 71 | Exon 6 | 2 | c.707C>A | p.Thr236Asn | (Li et al., 2018) | China. |
| 72 | Exon 6 | 1 | c.727C>T | p.Gln243Ter | (De Lucca & Casique, 2004) | Venezuela. |
| 73 | Intron 6 | 1 | c.737-1G>C | p.= | (Kruger et al., 2003) | USA. |
| 74 | Exon 7 | 8 | c.770C>T | p.Thr257Met | (Lee et al., 2005; Li et al., 2018; Poloni et al., 2018; Sebastio et al., 1995; Urreizti et al., 2006; Zaidi et al., 2012) | Brazil; China; Italy; Korea; Spain; Sudan. |
| 75 | Exon 7 | 5 | c.785C>T | p.Thr262Met | (Gallagher et al., 1998; Kim et al., 1997; Moat et al., 2004) | Ireland; Norway; USA. |
| 76 | Exon 7 | 4 | c.785C>G | p.Thr262Arg | (Gat-Yablonski et al., 2000; Guttormsen et al., 2001) | Israel; Norway. |
| 77 | Exon 7 | 11 | c.797G>A | p.Arg266Lys | (Guttormsen et al., 2001; Kim et al., 1997) | Norway. |
| 78 | Exon 7 | 2 | c.796A>G | p.Arg266Gly | (Katsushima et al., 2006) | Japan. |
| 79 | Exon 7 | 2 | c.824G>A | Cys275Tyr | (Cozar et al., 2011; Urreizti et al., 2003) | Spain. |
| 80 | Intron 7 | 10 | c.828+1G>A | p.(=) | (Janosík et al., 2001; Maclean et al., 2002; (Gaustadnes et al., 2002) Urreizti et al., 2006; Poloni et al., 2018) | Argentina; Australia; Brazil; Czech Republic; Dennmark. |
| 81 | Exon 8 | 133 | c.833T>C | p.Ile278Thr | (Cozar et al., 2011; Gallagher et al., 1998; Gallagher et al., 1995; Gat-Yablonski et al., 2000; Gaustadnes et al., 1998; Gaustadnes et al., 2002; Hu et al., 1993; Janosík et al., 2001; Katsushima et al., 2006; Kim et al., 1997; Kluijtmans et al., 1999; Kozich et al., 1997; Kruger et al., 2003; Kwok et al., 2011; Lefaucheur, Triquenot-Bagan, Quillard, Genevois, & Hannequin, 2008; Maclean et al., 2002; Moat et al., 2004; Orendáè et al., 2004; Poloni et al., 2018; Porto et al., 2005; Sebastio et al., 1995; Shih et al., 1995; Sperandeo et al., 1995; Voskoboeva et al., 2018) | Australia; Brazil; China; Czech Republic and Slovakia; Denmark; England; France; Germany; Ireland; Israel; Italy; Japan; Norway; Poland; Portugal; Russia; Spain; The Netherlands; USA. |
| 82 | Exon 8 | 1 | c.833T>G | p.Ile278Ser | (Cozar et al., 2011) | Spain. |
| 83 | Exon 8 | 1 | c.841G>A | p.Asp281Asn | (Cozar et al., 2011) | Spain. |
| 84 | Exon 8 | 2 | c.862G>A | p.Ala288Thr | (Bermúdez et al., 2006; Lee et al., 2005) | Colombia; Korea. |
| 85 | Exon 8 | 4 | c.864_868del | p.(Glu289Glyfs*39) | (Cozar et al., 2011; Poloni et al., 2018) | Brazil; Portugal. |
| 86 | Exon 8 | 1 | c.869C>T | p.Pro290Leu | (Sperandeo et al., 1995) | Spain. |
| 87 | Exon 8 | 1 | c.892dupC | p.(Gln298Profs*32) | (Gaustadnes et al., 2002) | Australia. |
| 88 | Exon 8 | 2 | c.904G>A | p. Glu302Lys | (Gaustadnes et al., 2002; Voskoboeva et al., 2018) | Australia; Russia. |
| 89 | Exon 8 | 2 | c.913G>A | p. Gly305Arg | (Voskoboeva et al., 2018) | Russia. |
| 90 | Exon 8 | 108 | c.919G>A | p.Gly307Ser | (de Franchis et al., 1999; Gallagher et al., 1998; Gaustadnes et al., 2002; Guttormsen et al., 2001; Hu et al., 1993; Kim et al., 1997; Kruger et al., 2003; Moat et al., 2004; Zschocke et al., 2009) | Australia; England; France; Germany; Ireland; Israel; Norway; Qatar; USA. |
| 91 | Exon 9 | 6 | c.959T>C | p.Val320Ala | (Guttormsen et al., 2001; Kim et al., 1997; Kruger et al., 2003) | Norway; USA |
| 92 | Exon 9 | 1 | c.962A>T | p.Asp321Val | (Cozar et al., 2011) | Argentina. |
| 93 | Exon 9 | 25 | c.969G>A | p.Trp323Ter | (Poloni et al., 2018; Zaidi et al., 2012) | Brazil; Saudi Arabia. |
| 94 | Exon 9 | 1 | c.989_  991delAGG | p.(Glu330del) | (Poloni et al., 2018) | Brazil. |
| 95 | Exon 9 | 149 | c.1006C>T | p.Arg336Cys | (de Franchis et al., 1999; El-Said et al., 2006; Gaustadnes et al., 2002; Kwok et al., 2011; Lee et al., 2005; Urreizti et al., 2003; Zaidi et al., 2012; Zschocke et al., 2009) | Australia; China; England; Korea; Portugal; Qatar; Spain; Saudi Arabia. |
| 96 | Exon 9 | 2 | c.1007G>A | p.Arg336His | (Coudé et al., 1998) | North Africa. |
| 97 | Exon 9 | 1 | c.1010dup | p.(Met337Ilefs*115) | (Li et al., 2018) | China. |
| 98 | Exon 9 | 4 | c.1013T>C | p.Leu338Pro | (Urreizti et al., 2006; Urreizti et al., 2003) | Spain. |
| 99 | Exon 9 | 3 | c.1039G>A | p.Gly347Ser | (Katsushima et al., 2006; Lee et al., 2005) | Japan; Korea. |
| 100 | Intron 9 | 2 | c.1039+1G>T | p.(=) | (Orendáè et al., 2004) | Poland. |
| 101 | Exon 10 | 3 | c.1046G>A | p.Ser349Asn | (Urreizti et al., 2003) | Spain. |
| 102 | Exon 10 | 12 | c.1058C>T | p.Thr353Met | (Cozar et al., 2011; Gaustadnes et al., 2002; Kruger et al., 2003; Lee et al., 2005; Poloni et al., 2018; Urreizti et al., 2006; Voskoboeva et al., 2018) | Australia; Brazil; Korea; Russia; Spain; USA. |
| 103 | Exon 10 | 1 | c.1060G>A | p.Val354Met | (Coudé et al., 1998) | Portugal. |
| 104 | Exon 10 | 1 | c.1063G>C | p. Ala355Pro | (Gallagher et al., 1998) | Ireland. |
| 105 | Exon 10 | 2 | c.1081G>A | p. Ala361Thr | (Castro et al., 2001) | Portugal. |
| 106 | Exon 10 | 1 | c.1102C>T | p.Gln368Ter | (Voskoboeva et al., 2018) | Russia. |
| 107 | Exon 10 | 2 | c.1105C>T | Arg369Cys | (Kim et al., 1997; Kluijtmans et al., 1999) | Norway; The Netherlands. |
| 108 | Exon 10 | 3 | c.1111G>A | p.Val371Met | (Gaustadnes et al., 2002; Kluijtmans et al., 1999) | Australia; The Netherlands. |
| 109 | Exon 10 | 4 | c.1126G>A | p. Asp376Asn | (Kruger et al., 2003; Poloni et al., 2018) | Brazil; USA. |
| 110 | Exon 10 | 4 | c.1136G>A | p.Arg379Gln | (Cozar et al., 2011; Urreizti et al., 2006; Urreizti et al., 2003) | Spain. |
| 111 | Exon 11 | 1 | c.1152G>C | p.Lys384Asn | (Voskoboeva et al., 2018) | Russia. |
| 112 | Exon 11 | 2 | c.1150A>G | p.Lys384Glu | (Aral et al., 1997) | France. |
| 113 | Exon 11 | 1 | c.1221delC | p.(Trp408Glyfs*16) | (Gaustadnes et al., 2002) | Australia. |
| 114 | Intron 11 | 1 | c.1223+1G>A | p.(=) | (Gaustadnes et al., 2002) | Australia. |
| 115 | Intron 11 | 20 | c.1224-2A>C | p.(=) | (Kozich et al., 1997; Janosík et al., 2001; Moat et al., 2004; Orendáè et al., 2004; Voskoboeva et al., 2018) | Czech Republic and Slovakia; Poland; Russia; USA |
| 116 | Intron 11 | 1 | c.1223+39del99 | p.? | (Janosík et al., 2001) | Czech Republic. |
| 117 | Intron 11 | 2 | c.1223+5G>T | p.(=) | (Poloni et al., 2018) | Brazil. |
| 118 | Exon 12 | 3 | c.1226G>A | p.Trp409Ter | (Janosík et al., 2001; Kozich et al., 1997) | Czech Republic and Slovakia. |
| 119 | Exon 12 | 1 | c.1259C>G | p.Ser420Ter | (Coudé et al., 1998) | North Africa. |
| 120 | Exon 12 | 1 | c.1265C>T | p.Pro422Leu | (Maclean et al., 2002) | Denmark. |
| 121 | Exon 12 | 1 | c.1286_1288delTCA | p.(Ile429del) | (Urreizti et al., 2006) | Argentina. |
| 122 | Exon 12 | 1 | c.1301C>A | p.Thr434Asn | (Kluijtmans et al., 1999) | The Netherlands. |
| 123 | Exon 12 | 1 | c.1304T>C | p.Ile435Thr | (Maclean et al., 2002) | Denmark. |
| 124 | Exon 12 | 4 | c.1321A>T | p.Lys441Ter | (Katsushima et al., 2006) | Japan. |
| 125 | Exon 12 | 16 | c.1330G>A | p.Asp444Asn | (Cozar et al., 2011; De Lucca & Casique, 2004; Kluijtmans et al., 1999; Lefaucheur et al., 2008; Maclean et al., 2002; Moat et al., 2004; Urreizti et al., 2006) | Denmark; France; Spain; The Netherlands; USA; Venezuela. |
| 126 | Exon 12 | 2 | c.1330G>T | p.Asp444Tyr | (Voskoboeva et al., 2018) | Russia. |
| 127 | Exon 12 | 1 | c.1336G>T | p. Ala446Ser | (Cozar et al., 2011) | Argentina. |
| 128 | Exon 13 | 2 | c.1367T>C | p.Leu456Pro | (Urreizti et al., 2003) | Spain. |
| 129 | Exon 14 | 1 | c.1471C>T | p.Arg491Cys | (Kluijtmans et al., 1999) | The Netherlands. |
| 130 | Exon 13 | 1 | c.1397C>T | p.Ser466Leu | (Maclean et al., 2002) | Denmark. |
| 131 | Exon 14 | 1 | c.1477dupA | p.(Thr493Asnfs*46) | (Li et al., 2018) | China. |
| 132 | Exon 14 | 1 | c.1498delT | p.(Ser500Argfs*41) | (Voskoboeva et al., 2018) | Russia. |
| 133 | Intron 14 | 1 | c.1553-1G>C | p.(=) | (Li et al., 2018) | China. |
| 134 | Exon 16 | 1 | c.1560_1569del | p.(Thr521Profs*17) | (Voskoboeva et al., 2018) | Russia. |
| 135 | Exon 16 | 10 | c.1566delG | p.(Lys523Serfs*18) | (Castro et al., 2001; Urreizti et al., 2003; Cozar et al., 2011) | Portugal; Spain. |
| 136 | Exon 16 | 1 | c.1576C>A | p. Gln526Lys | (Kruger et al., 2003) | USA. |
| 137 | Exon 16 | 1 | c.1591_1594del | p.(Phe531Glyfs*9) | (Katsushima et al., 2006) | Japan. |
| 138 | Exon 16 | 2 | c.1616T>C | p.Leu539Ser | (Aral et al., 1997) | France. |
| 139 | Exon 16 | 1 | c.1622_1623insTGGA | p.(Phe542Glyfs*37) | (Gaustadnes et al., 2002) | Australia. |
| 140 | Exon 16 | 3 | c.1627_1645del | p.(Val543Thrfs*26) | (Gat-Yablonski et al., 2000) | Israel. |

Note: 159 alleles with no pathogenic variant identified.

**REFERENCES**

Aral, B., Coudé, M., London, J., Aupetit, J., Chassé, J. F., Zabot, M. T., Kamoun, P. (1997). Two novel mutations (K384E and L539S) in the C-terminal moiety of the cystathionine beta-synthase protein in two French pyridoxine-responsive homocystinuria patients. *Hum Mutat, 9*(1), 81-82. doi:10.1002/(SICI)1098-1004(1997)9:1<81::AID-HUMU18>3.0.CO;2-L

Bermúdez, M., Frank, N., Bernal, J., Urreizti, R., Briceño, I., Merinero, B., Kraus, J. P. (2006). High prevalence of CBS p.T191M mutation in homocystinuric patients from Colombia. *Hum Mutat, 27*(3), 296. doi:10.1002/humu.9416

Castro, R., Heil, S. G., Rivera, I., Jakobs, C., de Almeida, I. T., & Blom, H. J. (2001). Molecular genetic analysis of the cystathionine beta-synthase gene in Portuguese homocystinuria patients: three novel mutations. *Clin Genet, 60*(2), 161-163.

Coudé, M., Aupetit, J., Zabot, M. T., Kamoun, P., & Chadefaux-Vekemans, B. (1998). Four novel mutations at the cystathionine beta-synthase locus causing homocystinuria. *J Inherit Metab Dis, 21*(8), 823-828.

Cozar, M., Urreizti, R., Vilarinho, L., Grosso, C., Dodelson de Kremer, R., Asteggiano, C. G., Balcells, S. (2011). Identification and functional analyses of CBS alleles in Spanish and Argentinian homocystinuric patients. *Hum Mutat, 32*(7), 835-842. doi:10.1002/humu.21514

de Franchis, R., Kraus, E., Kozich, V., Sebastio, G., & Kraus, J. P. (1999). Four novel mutations in the cystathionine beta-synthase gene: effect of a second linked mutation on the severity of the homocystinuric phenotype. *Hum Mutat, 13*(6), 453-457. doi:10.1002/(SICI)1098-1004(1999)13:6<453::AID-HUMU4>3.0.CO;2-K

De Lucca, M., & Casique, L. (2004). Characterization of cystathionine beta-synthase gene mutations in homocystinuric Venezuelan patients: identification of one novel mutation in exon 6. *Mol Genet Metab, 81*(3), 209-215. doi:10.1016/j.ymgme.2003.12.003

El-Said, M. F., Badii, R., Bessisso, M. S., Shahbek, N., El-Ali, M. G., El-Marikhie, M., Zschocke, J. (2006). A common mutation in the CBS gene explains a high incidence of homocystinuria in the Qatari population. *Hum Mutat, 27*(7), 719. doi:10.1002/humu.9436

Gallagher, P. M., Naughten, E., Hanson, N. Q., Schwichtenberg, K., Bignell, M., Yuan, M., Tsai, M. Y. (1998). Characterization of mutations in the cystathionine beta-synthase gene in Irish patients with homocystinuria. *Mol Genet Metab, 65*(4), 298-302. doi:10.1006/mgme.1998.2771

Gallagher, P. M., Ward, P., Tan, S., Naughten, E., Kraus, J. P., Sellar, G. C., Whitehead, A. S. (1995). High frequency (71%) of cystathionine beta-synthase mutation G307S in Irish homocystinuria patients. *Hum Mutat, 6*(2), 177-180. doi:10.1002/humu.1380060211

Gat-Yablonski, G., Mandel, H., Fowler, B., Taleb, O., & Sela, B. A. (2000). Homocystinuria in the Arab population of Israel: identification of two novel mutations using DGGE analysis. *Hum Mutat, 16*(4), 372. doi:10.1002/1098-1004(200010)16:4<372::AID-HUMU12>3.0.CO;2-J

Gaustadnes, M., Kluijtmans, L. A., Jensen, O. K., Rasmussen, K., Heil, S. G., Kraus, J. P., Rüdiger, N. (1998). Detection of a novel deletion in the cystathionine beta-synthase (CBS) gene using an improved genomic DNA based method. *FEBS Lett, 431*(2), 175-179.

Gaustadnes, M., Wilcken, B., Oliveriusova, J., McGill, J., Fletcher, J., Kraus, J. P., & Wilcken, D. E. (2002). The molecular basis of cystathionine beta-synthase deficiency in Australian patients: genotype-phenotype correlations and response to treatment. *Hum Mutat, 20*(2), 117-126. doi:10.1002/humu.10104

Gong, B., Liu, L., Li, Z., Ye, Z., Xiao, Y., Zeng, G., Yang, Z. (2015). Novel Compound Heterozygous CBS Mutations Cause Homocystinuria in a Han Chinese Family. *Sci Rep, 5*, 17947. doi:10.1038/srep17947

Guttormsen, A. B., Ueland, P. M., Kruger, W. D., Kim, C. E., Ose, L., Følling, I., & Refsum, H. (2001). Disposition of homocysteine in subjects heterozygous for homocystinuria due to cystathionine beta-synthase deficiency: relationship between genotype and phenotype. *Am J Med Genet, 100*(3), 204-213.

Hu, F. L., Gu, Z., Kozich, V., Kraus, J. P., Ramesh, V., & Shih, V. E. (1993). Molecular basis of cystathionine beta-synthase deficiency in pyridoxine responsive and nonresponsive homocystinuria. *Hum Mol Genet, 2*(11), 1857-1860.

Janosík, M., Oliveriusová, J., Janosíková, B., Sokolová, J., Kraus, E., Kraus, J. P., & Kozich, V. (2001). Impaired heme binding and aggregation of mutant cystathionine beta-synthase subunits in homocystinuria. *Am J Hum Genet, 68*(6), 1506-1513. doi:10.1086/320597

Katsushima, F., Oliveriusova, J., Sakamoto, O., Ohura, T., Kondo, Y., Iinuma, K., Kraus, J. P. (2006). Expression study of mutant cystathionine beta-synthase found in Japanese patients with homocystinuria. *Mol Genet Metab, 87*(4), 323-328. doi:10.1016/j.ymgme.2005.09.013

Kim, C. E., Gallagher, P. M., Guttormsen, A. B., Refsum, H., Ueland, P. M., Ose, L., Kruger, W. D. (1997). Functional modeling of vitamin responsiveness in yeast: a common pyridoxine-responsive cystathionine beta-synthase mutation in homocystinuria. *Hum Mol Genet, 6*(13), 2213-2221.

Kluijtmans, L. A., Boers, G. H., Kraus, J. P., van den Heuvel, L. P., Cruysberg, J. R., Trijbels, F. J., & Blom, H. J. (1999). The molecular basis of cystathionine beta-synthase deficiency in Dutch patients with homocystinuria: effect of CBS genotype on biochemical and clinical phenotype and on response to treatment. *Am J Hum Genet, 65*(1), 59-67. doi:10.1086/302439

Kozich, V., Janosík, M., Sokolová, J., Oliveriusová, J., Orendác, M., Kraus, J. P., & Elleder, D. (1997). Analysis of CBS alleles in Czech and Slovak patients with homocystinuria: report on three novel mutations E176K, W409X and 1223 + 37 del99. *J Inherit Metab Dis, 20*(3), 363-366.

Kruger, W. D., Wang, L., Jhee, K. H., Singh, R. H., & Elsas, L. J. (2003). Cystathionine beta-synthase deficiency in Georgia (USA): correlation of clinical and biochemical phenotype with genotype. *Hum Mutat, 22*(6), 434-441. doi:10.1002/humu.10290

Kwok, J. S., Fung, S. L., Lui, G. C., Law, E. L., Chan, M. H., Leung, C. B., & Tang, N. L. (2011). CBS gene mutations found in a Chinese pyridoxine-responsive homocystinuria patient. *Pathology, 43*(1), 81-83. doi:10.1097/PAT.0b013e3283419dbb

Lee, S. J., Lee, D. H., Yoo, H. W., Koo, S. K., Park, E. S., Park, J. W., Jung, S. C. (2005). Identification and functional analysis of cystathionine beta-synthase gene mutations in patients with homocystinuria. *J Hum Genet, 50*(12), 648-654. doi:10.1007/s10038-005-0312-2

Lefaucheur, R., Triquenot-Bagan, A., Quillard, M., Genevois, O., & Hannequin, D. (2008). [Stroke and iridodonesis revealing a homocystinuria caused by a compound heterozygous mutation of cystathionine beta-synthase]. *Rev Neurol (Paris), 164*(8-9), 728-732. doi:10.1016/j.neurol.2007.12.004

Li, D. X., Li, X. Y., Dong, H., Liu, Y. P., Ding, Y., Song, J. Q., Yang, Y. L. (2018). Eight novel mutations of CBS gene in nine Chinese patients with classical homocystinuria. *World J Pediatr, 14*(2), 197-203. doi:10.1007/s12519-018-0135-9

Maclean, K. N., Gaustadnes, M., Oliveriusová, J., Janosík, M., Kraus, E., Kozich, V., Kraus, J. P. (2002). High homocysteine and thrombosis without connective tissue disorders are associated with a novel class of cystathionine beta-synthase (CBS) mutations. *Hum Mutat, 19*(6), 641-655. doi:10.1002/humu.10089

Marble, M., Geraghty, M. T., de Franchis, R., Kraus, J. P., & Valle, D. (1994). Characterization of a cystathionine beta-synthase allele with three mutations in cis in a patient with B6 nonresponsive homocystinuria. *Hum Mol Genet, 3*(10), 1883-1886.

Moat, S. J., Bao, L., Fowler, B., Bonham, J. R., Walter, J. H., & Kraus, J. P. (2004). The molecular basis of cystathionine beta-synthase (CBS) deficiency in UK and US patients with homocystinuria. *Hum Mutat, 23*(2), 206. doi:10.1002/humu.9214

Orendáè, M., Pronicka, E., Kubalska, J., Janosik, M., Sokolová, J., Linnebank, M., Kozich, V. (2004). Identification and functional analysis of two novel mutations in the CBS gene in Polish patients with homocystinuria. *Hum Mutat, 23*(6), 631. doi:10.1002/humu.9249

Poloni, S., Sperb-Ludwig, F., Borsatto, T., Weber Hoss, G., Doriqui, M. J. R., Embiruçu, E. K., Schwartz, I. V. D. (2018). CBS mutations are good predictors for B6-responsiveness: A study based on the analysis of 35 Brazilian Classical Homocystinuria patients. *Mol Genet Genomic Med*. doi:10.1002/mgg3.342

Porto, M. P., Galdieri, L. C., Pereira, V. G., Vergani, N., da Rocha, J. C., Micheletti, C., Almeida, V. D. (2005). Molecular analysis of homocystinuria in Brazilian patients. *Clin Chim Acta, 362*(1-2), 71-78. doi:10.1016/j.cccn.2005.05.030

Sebastio, G., Sperandeo, M. P., Panico, M., de Franchis, R., Kraus, J. P., & Andria, G. (1995). The molecular basis of homocystinuria due to cystathionine beta-synthase deficiency in Italian families, and report of four novel mutations. *Am J Hum Genet, 56*(6), 1324-1333.

Shih, V. E., Fringer, J. M., Mandell, R., Kraus, J. P., Berry, G. T., Heidenreich, R. A., Ramesh, V. (1995). A missense mutation (I278T) in the cystathionine beta-synthase gene prevalent in pyridoxine-responsive homocystinuria and associated with mild clinical phenotype. *Am J Hum Genet, 57*(1), 34-39.

Sperandeo, M. P., Panico, M., Pepe, A., Candito, M., de Franchis, R., Kraus, J. P., Sebastio, G. (1995). Molecular analysis of patients affected by homocystinuria due to cystathionine beta-synthase deficiency: report of a new mutation in exon 8 and a deletion in intron 11. *J Inherit Metab Dis, 18*(2), 211-214.

Urreizti, R., Asteggiano, C., Bermudez, M., Córdoba, A., Szlago, M., Grosso, C., Grinberg, D. (2006). The p.T191M mutation of the CBS gene is highly prevalent among homocystinuric patients from Spain, Portugal and South America. *J Hum Genet, 51*(4), 305-313. doi:10.1007/s10038-006-0362-0

Urreizti, R., Balcells, S., Rodés, M., Vilarinho, L., Baldellou, A., Couce, M. L., Grinberg, D. (2003). Spectrum of CBS mutations in 16 homocystinuric patients from the Iberian Peninsula: high prevalence of T191M and absence of I278T or G307S. *Hum Mutat, 22*(1), 103. doi:10.1002/humu.9153

Voskoboeva, E., Semyachkina, A., Yablonskaya, M., & Nikolaeva, E. (2018). Homocystinuria due to cystathionine beta-synthase (CBS) deficiency in Russia: Molecular and clinical characterization. *Mol Genet Metab Rep, 14*, 47-54. doi:10.1016/j.ymgmr.2017.11.001

Zaidi, S. H., Faiyaz-Ul-Haque, M., Shuaib, T., Balobaid, A., Rahbeeni, Z., Abalkhail, H., Al-Owain, M. (2012). Clinical and molecular findings of 13 families from Saudi Arabia and a family from Sudan with homocystinuria. *Clin Genet, 81*(6), 563-570. doi:10.1111/j.1399-0004.2011.01690.x

Zschocke, J., Kebbewar, M., Gan-Schreier, H., Fischer, C., Fang-Hoffmann, J., Wilrich, J., Hoffmann, G. F. (2009). Molecular neonatal screening for homocystinuria in the Qatari population. *Hum Mutat, 30*(6), 1021-1022. doi:10.1002/humu.20994
